# Suicidal ideation and suicide attempts among students aged 12 to 24 after the lifting of COVID-19 restrictions in China: prevalence and associated factors

**DOI:** 10.3389/fpsyt.2024.1383992

**Published:** 2024-06-18

**Authors:** Qing-Qing Xiao, Xue-Hua Huang, Jing Yang, Yun-Fei Mu, Cong Wang, Zhong-Yue Deng, Jia Cai, Ai-Ping Deng, Wan-Jie Tang, Xia-Can Chen, Wei Shi, Yi Jiang, Jia-Jun Xu, Li Yin, Yi Huang, Wei Zhang, Mao-Sheng Ran

**Affiliations:** ^1^ Mental Health Center, West China Hospital, Sichuan University, Chengdu, Sichuan, China; ^2^ West China School of Nursing, Sichuan University, Chengdu, Sichuan, China; ^3^ Institute of Psychiatry, West China Hospital, Sichuan University, Chengdu, Sichuan, China; ^4^ Institute of Forensic Medicine, West China School of Basic Medical Sciences and Forensic Medicine, Sichuan University, Chengdu, Sichuan, China; ^5^ Institute for Disaster Management and Reconstruction, Sichuan University, Chengdu, Sichuan, China; ^6^ Department of Neurology, West China Hospital, Sichuan University, Chengdu, Sichuan, China

**Keywords:** suicidal ideation, suicide attempts, adolescent and young adults, lifting of COVID-19 restrictions, prevalence, associated factors

## Abstract

**Objective:**

To investigate the prevalence and associated factors of suicidal ideation and suicide attempts among adolescent and young adults in China from December 14, 2022 to February 28, 2023, when COVID-19 restrictions were lifted.

**Methods:**

Students in middle and high schools and colleges and universities in the province of Sichuan, China were asked to complete on-line cross-sectional surveys. Information was collected about sociodemographics, experiences related to the COVID-19 pandemic, suicidal ideation and suicide attempts. Participants also filled out the Patient Health Questionnaire-9, the Generalized Anxiety Disorder-7 and the Social Support Rate Scale surveys. Factors associated with suicidal ideation or suicide attempts were explored using logistic regression.

**Results:**

Of the 82,873 respondents (aged 12 to 24 years), 21,292 (25.7%) reported having thought of suicide at least once in their lifetime, 10,382 (12.5%) reported having thought about suicide within the previous 12 months, and 1,123 (1.4%) reported having attempted it within the previous 12 months. Risk of lifetime suicidal ideation was higher among middle school students than among older students. Risk of suicidal ideation and risk of suicide attempts correlated directly with severity of symptoms of depression and anxiety, and inversely with level of social support. Greater risk of suicidal ideation and suicidal attempts was associated with: being female, living in an urban environment, attending a boarding school, currently being in love, having parents who divorced or remarried, having parents who exhibit non-authoritative parenting behavior, having higher family income, having been COVID-19 infected, having been quarantined for a long time, and being dissatisfied with one’s education.

**Conclusions:**

Suicidal ideation and suicide attempts remain prevalent among young people in China. The potential associated factors identified in our study may be useful for targeting appropriate psychosocial interventions and developing mental health policies.

## Introduction

Mental health problems are common in young people. In 2019, the global the reported mean prevalence of mental disorders in 5- to 24-year-olds was 11.63% (1). The prevalence of mental disorders in the age 5 to 9 years group was estimated at approximately half (6.81%; 95% UI, 5.58–8.03) the rate recorded for the age 20 to 24 years group (13.63%; 95% UI, 11.90–15.53) (1). The steep increase in mood disorders across early to late adolescence is particularly striking (1).

Also concerning is the notable increase in suicidal thoughts, attempts, and competitions that emerges in adolescents (2). In fact, suicide is the third leading cause of injury-related death among adolescents (3), and in China, it is one of the most frequent causes of death among people aged 15–34 years (4). The prevalence of suicidal ideation, defined as thoughts of harming or killing oneself actively or passively (5), ranges from 17.7% to 23.5% among adolescents with a mean age of 15 ± 1 years in China (6) which compares to 18.8% in the US (7). In a survey of students aged 10–20 years in four provinces in China between 2017 and 2018 (8), 24.6% reported suicidal ideation during the previous 12 months, while 12.0% reporting having planned suicide and 4.9% reported attempting suicide. A study of more than a quarter million adolescents aged 11–17 years across 77 countries from the 2020 Global School-based Student Health Survey (GSHS) indicated population-weighted prevalences of 18% for suicidal ideation and 16% for suicide attempts during the previous 12 months (9). Given these high rates, it is not surprising that suicide is a global public health concern among young people (10).

Although the literature clearly indicates high prevalence of suicidal ideation and suicide attempts around the world, how these prevalences vary during developmental stages, from early adolescence through to early adulthood, is poorly understood. To address this gap in knowledge, we survey a large population of students aged 12 to 24. Data were collected between December 14, 2022 to February 28, 2023, when COVID-19 restrictions were lifted. During the COVID-19 pandemic there were three phases of pandemic response in China (11): 1) nation-wide lockdown (January 1, 2020 to April 29, 2020); 2) routine infection prevention and control (April 30, 2020 to December 6, 2022); and 3) lifting of COVID-19 re strictions (after December 7, 2022).

Substantial evidence indicates that the pandemic and associated biosafety measures, including lockdowns, home confinement, self-isolation, and social distancing (12, 13), have contributed to a rise in mental health problems among adolescents, such as sleep problems, anxiety, depression, and suicidality (14). A two-wave longitudinal web-based survey revealed that 20.0% of high school students aged 12 to 18 reported suicidal ideation or suicide attempts between July 11 and July 23, 2020 (15). In another Chinese study, 19.6% of university students endorsed suicidal ideation between March 20th and April 10th 2020 (16). A study on high school students in China during nation-wide lockdown showed that 31.3% and 7.5% of students reported suicidal ideation and attempts, respectively (17). Taken together, the prevalence of suicide ideation and suicide attempt among children and adolescents increased during the COVID-19 pandemic compared with that before the pandemic. In a meta-analysis of 11.1 million emergency department (ED) visits for any health issue, an increase of 22% in ED visits for suicide attempts was found during the pandemic. The increase for suicide attempts was particularly pronounced for teenage girls (39% vs. 6% for teenage boys) (18).

Even though essentially all COVID-19 restrictions were lifted in China in December 2022 and even earlier in other parts of the world, the continuing risk of COVID-19 infection, distress around such infection and its consequences, and the financial consequences of the pandemic on employment and family businesses may have affected mental health (19–21), and suicidality in particular. This may be especially true for adolescents and young adults, when their social and learning need to belong is strongest, but when this need was restricted due to COVID-19 pandemic (22).

The psychosocial factors affecting adolescent suicide are complex. A previous review identified three main factors that appear to increase the risk of suicidality: psychological factors (depression, anxiety, and previous suicide attempt); stressful life events (family problems and peer conflicts); and personality traits (such as neuroticism and impulsivity) (23). Another study found the factors that increased the attempted suicide possibility: included being a woman, being under 16 years of age, living in an unfavorable family conditions or having a poor communication with parents, having low self-esteem, behavioral or learning problems or having a mental disorder; or having been forced into sexual contact. A cross-sectional study also found that early romantic experiences and sexual behavior were associated with poorer adolescent health and well-being outcomes (24). In Chinese adolescents, sexual violence was found to be related to suicide ideation (25). Suicide is a multidimensional and multicausal problem associated with social determinants of health (26). As a result, its incidence and influencing factors need to be further explored, especially after the lifting of COVID-19 restrictions.

In the present study we surveyed students from middle schools, high schools, colleges and universities in the province of Sichuan, China to assess the prevalence of suicidal ideation and suicide attempts after the lifting of COVID-19 restrictions. In addition, we examined associated factors that might predict risk of such suicidal behavior in the aftermath of the pandemic. These findings contribute to our understanding of suicide behavior among adolescents.

## Methods

### Study population

An online questionnaire was prepared using the Wenjuanxing platform (www.wjx.cn) and sent to a convenience sample of school principals or teachers at middle schools, high schools, colleges and universities in Sichuan province, China. The principals and teachers were asked to forward the questionnaire to their students, who were invited to complete the survey from December 14, 2022 to February 28, 2023. To access the questionnaire, students had to provide informed consent for their anonymized responses to be analyzed and published for research purposes.

Students were included in the study if they were 12–24 years old and able to read and understand the questionnaire. Students were excluded if they had difficulty completing the questionnaire. For example, without a mobile phone, or it is not possible to answer all questions. Of the 90,118 students who began to fill it out, 82,873 (92.0%) provided informed consent and completed the survey, while 7245 (8.0%) did not. The overall study was approved by the Biomedical Research Ethics Committee at West China Hospital of Sichuan University (2022–1790).

### Questionnaire

The survey had four parts and required 15–25 minutes to complete. The first part collected data on sociodemographic characteristics, including gender, age, type of registered residence (rural or urban), currently being in love, marital status, household family income level, and parenting style. The second part asked about the students’ experiences during the COVID-19 pandemic, including history of COVID-19 infection, time spent in quarantine, home-based study, impact of the pandemic on academic performance, whether their studies had returned to normal, satisfaction with his or her own academic performance, impact of the pandemic on future study or employment, psychological knowledge learning, and concern about COVID-19 infection in the future.

The third part of the questionnaire integrated three international instruments for evaluating symptoms of depression and anxiety, as well as level of social support. The Patient Health Questionnaire-9 (27) was used to assess depression as “none” (scores of 0–4), “mild” (scores of 5–9), “moderate” (scores of 10–14), or “severe” (scores of 15–27). This survey has shown good psychometric performance in samples of Chinese adolescents (28). The Generalized Anxiety Disorder-7 (29) was used to categorize the severity of possible anxiety, based on symptoms during the previous two weeks, as “none” (scores 0–4), “mild” (scores 5–9), “moderate” (scores of 10–14) or “severe” (scores of 15–21). This survey has shown Cronbach’s α of 0.92 on a Chinese sample (30). The Social Support Rate Scale (31) was used to categorize an individual’s level of social support as “low” (scores ≤ 22), “medium” (scores of 23–44) or “high” (scores of 45–66) (32). This survey has shown good validity and reliability in multiple Chinese samples (33, 34).

The fourth part of the questionnaire asked “Have you ever thought of committing suicide in your life?” to which the respondent could answer “Yes” or “No”; and “Have you had any suicidal thoughts or behaviors in the past 12 months?” to which the respondent could answer “Yes, have had suicidal thoughts”, “Yes, have had suicidal behaviors”, or “No”. These two questions can be effective for assessing suicidal ideation and suicide attempts (35–37).

### Statistical analysis

Data were analyzed statistically using SPSS 21.0 (IBM, Armonk, NY, USA), and results associated with a two-sided *P* < 0.05 were considered statistically significant. Differences between respondents who reported ever having thought about suicide or not were assessed for significance using Pearson chi-squared tests. Differences among those who had thought about suicide, attempted suicide, or neither within the previous 12 months were assessed using Pearson chi-squared tests. Factors that in univariate analysis were significantly associated with suicidal ideation or suicide attempts, as separate outcomes, were entered into logistic regression. In view of the large number of independent variables and large sample size in this study, logistic stepwise forward regression (Likelihood Ratio) method was used to help reduce false positive results. This approach corrects for variables with little contribution and for collinearity.

## Results

### Demographics of the sample

Of the 90,118 students at 162 middle or high schools, colleges and universities in Sichuan who began to fill out the questionnaire, 82,873 (92.0%) with a mean age of 16.97 ± 2.29 years (range, 12–24 years) completed it, of whom 35,386 (42.7%) were male (**Table 1**). Nearly half of participants (36,111, 43.6%) were high school students, while nearly equal proportions were attending middle school (24,157, 29.1%) or college or university (22,605, 27.3%).

**Table 1:** Demographic Characteristics and Epidemic-Related Information for the Total Sample.

Variables	Participants, No. (%)	Variables	Participants, No. (%)
**Gender**		**How did your study at home during the COVID-19**	
Male	35386 (42.7)	No learning	2335 (2.8)
Female	47487 (57.3)	Occasional learning	41354 (49.9)
**Registered residence**		Regular learning	39184 (47.3)
Rural area	67740 (81.7)	**Academic satisfaction during the COVID-19**	
Urban area	15133 (18.3)	Extremely satisfied	5 639 (6.8)
**School type**		Satisfied	13 617 (16.4)
Middle school	24157 (29.1)	General Satisfied	47 704 (57.6)
High school	36111 (43.6)	Dissatisfied	12 956 (15.6)
College or university	22605 (27.3)	Extremely dissatisfied	2 957 (3.6)
**Accommodation Type**		**Did your study returned to normal status**	
Day pupil	10731 (12.9)	No recovery	8977 (10.8)
Boarders at school	71428 (86.2)	Partial recovery	56761 (68.5)
Other	714 (0.9)	Full recovery	17135 (20.7)
**Love situation**		**Have you actively learned about mental health related knowledge after the COVID-19**	
Not in a romantic relationship	74442 (89.8)	Yes	50455 (60.9)
Having a boyfriend	4636 (5.6)	No	32418 (39.1)
Having a girlfriend	3209 (3.9)	**Does the epidemic have an impact on your further study and employment**	
Other	586 (0.7)	No impact	20523 (24.8)
**Family economic level**		Minor impact	34114 (41.2)
Higher than local average	1777 (2.1)	Significant impact	15360 (18.5)
local average	54186 (65.4)	Serious impact	4124 (5.0)
Below local average	26910 (32.5)	Unclear	8752 (10.6)
**Parents’ marital status**		**Worry about being infected**	
Unmarried	1 410 (1.7)	Not worried	21056 (25.4)
Married	66 948 (80.8)	A little worried	49271 (59.5)
Divorce	7 525 (9.1)	Quite worried	8925 (10.8)
Remarried	5 586 (6.7)	Extremely worried	3621 (4.4)
Other	1 404 (1.7)	**PHQ-9**	
**Parenting styles**		No depression	51309 (61.9)
Authoritative pattern	45 120 (54.4)	Mild depression	17792 (21.5)
Autocratic pattern	18 593 (22.4)	Moderate depression	6674 (8.1)
Neglectful pattern	6 378 (7.7)	Severe depression	7098 (8.6)
Submissive pattern	12 782 (15.4)	**Anxiety Symptoms**	
**Have you been infected with COVID-19**		No anxiety	56482 (68.2)
Yes	33314 (40.2)	Mild anxiety	16910 (20.4)
No	49559 (59.8)	Moderate anxiety	6718 (8.1)
**Quarantine time**		Severe anxiety	2763 (3.3)
0 days	58639 (70.8)	**Social support**	
<7 days	10536 (12.7)	Low-level	4264 (5.1)
7–14 days	10458 (12.6)	Medium-level	76843 (92.7)
>14 days	3240 (3.9)	High-level	1766 (2.1)

Most students (74,442, 89.8%) reported that they were not currently in love. More than half (54186, 65.4%) reported that their family’s household income was around the local average, while just over half (45,120, 54.4%) considered the parenting style at home to be authoritative, followed by autocratic (18,593, 22.4%), submissive (12,782, 15.4%) and neglectful (6,378, 7.7%). Fewer than half of participants (33,314, 40.2%) reported having been infected with the SARS-CoV-2 virus, and just over one quarter (24,234, 29.2%) reported having been quarantined.

### Suicidal ideation and suicide attempts among students

**Table 2** shows the prevalence of suicidal ideation and attempts in participants. Overall, 21292 participants (25.7%) reported lifetime suicidal ideation, 10382 participants (12.5%) had suicidal ideation in the past year, and 1123 participants (1.4%) had suicide attempts in the past year. The results showed that the lifetime suicidal ideation was more prevalent for participants who were female, middle and high school students, from urban area, with higher family economic level than local average, COVID-19 infection, with quarantine experience, with worse academic performance, and without learning mental health knowledge (*P*<0.05). Different levels of anxiety symptoms, depression symptoms, and social support were significantly related to the lifetime suicidal ideation (*P*<0.05). Most factors that affected lifetime suicidal ideation were also factors that affected 12-month suicidal ideation and attempts. But there were no statistically significant differences among the three factors of registered residence location, family economic level and academic performance in the 12-month suicidal ideation and attempts (*P*>0.05).

**Table 2:** Suicidal Ideation and Suicide Attempts in Students Stratified by Epidemic-Related Factors.

Variables	Lifetime Suicidal Ideation				12-month Suicidal Ideation or Suicide Attempts				
	Yes N (%)	No N (%)	χ^2^	*P*	Suicidal Ideation N (%)	Suicide Attempts N (%)	No	χ^2^	*P*
**Overall**	21292 (25.7)	61581 (74.3)			10382 (12.5)	1123 (1.4)	9787 (11.8)		
Gender									
Male	6941 (19.6)	28445 (80.4)	1194.656	0.000	3215 (9.1)	286 (0.8)	3440 (9.7)	67.534	0.000
Female	14351 (30.2)	33136 (69.8)			7167 (15.1)	837 (1.8)	6347 (13.4)		
Registered residence									
Rural area	17062 (25.2)	50678 (74.8)	49.523	0.000	8358 (12.3)	875 (1.3)	7829 (11.6)	4.486	0.106
Urban area	4230 (28.0)	10903 (72.0)			2024 (13.4)	248 (1.6)	1958 (12.9)		
School type									
Middle school	5121 (21.2)	19036 (78.8)	449.104	0.000	2760 (11.4)	428 (1.8)	1933 (8.0)	634.731	0.000
High school	10430 (28.9)	25681 (71.1)			5416 (15.0)	534 (1.5)	4480 (12.4)		
College or University	5741 (25.4)	16864 (74.6)			2206 (9.8)	161 (0.7)	3374 (14,9)		
Accommodation Type									
Day pupil	2479 (23.1)	8252 (76.9)	43.384	0.000	1326 (12.4)	169 (1.6)	984 (9.2)	55.307	0.000
Boarders at school	18629 (26.1)	52799 (74.0)			8977 (12.6)	938 (1.3)	8714 (12.2)		
Other	184 (25.8)	530 (74.2)			79 (11.1)	16 (2.2)	89 (12.5)		
Love situation									
Not in a romantic relationship	18749 (25.2)	55693 (74.8)	196.605	0.000	9203 (12.4)	958 (1.3)	8588 (11.5)	45.189	0.000
Having a boyfriend	1568 (33.8)	3068 (66.2)			720 (15.5)	85 (1.8)	763 (16.5)		
Having a girlfriend	775 (24.2)	2434 (75.8)			363 (11.3)	51 (1.6)	361 (11.2)		
Other	200 (34.1)	385 (65.7)			96 (16.4)	29 (4.9)	75 (12.8)		
Family economic level									
Higher than local average	537 (30.2)	1240 (69.8)	173.840	0.000	262 (14.7)	32 (1.8)	243 (13.7)	1.895	0.755
Local average	13140 (24.2)	41046 (75.8)			6392 (11.8)	676 (1.2)	6072 (11.2)		
Below local average	7615 (28.3)	19295 (71.7)			3728 (13.9)	415 (1.5)	3472 (12.9)		
Parental marriage									
Unmarried	388 (27.5)	1022 (72.5)	366.983	0.000	216 (15.3)	19 (1.3)	153 (10.9)	111.556	0.000
Married	16280 (24.3)	50668 (75.7)			7697 (11.5)	798 (1.2)	7785 (11.6)		
Divorce	2333 (31.0)	5192 (69.0)			1221 (16.2)	139 (1.8)	973 (12.9)		
Remarried	1804 (32.3)	3782 (67.7)			983 (17.6)	130 (2.3)	691 (12.4)		
Other	487 (34.7)	917 (65.3)			265 (18.9)	37 (2.6)	185 (13.2)		
Parenting style									
Authoritative pattern	9010 (20.0)	36110 (80.0)	2214.885	0.000	3971 (8.8)	394 (0.9)	4645 (10.3)	243.251	0.000
Autocratic pattern	6021 (32.4)	12572 (67.6)			3174 (17.1)	356 (1.9)	2491 (13.4)		
Neglective pattern	2731 (42.8)	3647 (57.2)			1494 (23.4)	196 (3.1)	1041 (16.3)		
Submissive pattern	3530 (27.6)	9252 (72.4)			1743 (13.6)	177 (1.4)	1610 (12.6)		
Have you been infected with COVID-19									
Yes	9962 (30.0)	23352 (70.1)	517.434	0.000	4980 (14.9)	539 (1.6)	4443 (13.3)	14.068	0.001
No	11330 (22.9)	38229 (77.1)			5402 (10.9)	584 (1.2)	5344 (10.8)		
Quarantine time									
0 days	14099 (24.0)	44540 (76.0)	372.587	0.000	6815 (11.6)	739 (1.3)	6545 (11.2)		
<7 days	2949 (28.0)	7587 (72.0)			1438 (48.8)	144 (4.9)	1367 (46.4)	15.906	0.014
7–14 days	3073 (29.6)	7385 (70.4)			1575 (50.7)	160 (5.3)	1338 (44.0)		
>14 days	1171 (36.1)	2069 (63.9)			554 (47.3)	80 (6.8)	537 (45.9)		
How did you study at home during the COVID-19									
No learning	888 (38.0)	1447 (62.0)	1054.974	0.000	501 (21.5)	47 (2.0)	340 (14.6)	26.789	0.000
Occasional learning	12298 (29.7)	29056 (70.3)			5996 (14.5)	668 (1.6)	5634 (13.6)		
Regular learning	8106 (20.7)	31078 (79.3)			3885 (9.9)	408 (1.0)	3813 (9.7)		
Academic during the COVID-19									
Academic progress	2636 (17.1)	12779 (82.9)	1141.295	0.000	1284 (8.3)	110 (0.7)	1242 (8.1)	9.466	0.050
Almost no impact	7835 (24.1)	24642 (75.9)			3780 (11.6)	423 (1.3)	3632 (11.2)		
Academic decline	10821 (30.9)	24160 (69.1)			5318 (15.2)	590 (1.7)	4913 (14.0)		
Academic satisfaction									
Extremely satisfied	1027 (18.2)	4612 (81.8)	2738.795	0.000	529 (9.4)	36 (0.6)	462 (8.2)	311.909	0.000
Satisfied	2161 (15.9)	11456 (84.1)			901 (6.6)	85 (0.6)	1175 (8.6)		
General Satisfied	11820 (24.8)	35884 (75.2)			5520 (11.6)	575 (1.2)	5725 (12.0)		
Dissatisfied	4759 (36.7)	8197 (63.3)			2504 (19.3)	290 (2.2)	1965 (15.2)		
Extremely dissatisfied	1525 (51.6)	1432 (48.4)			928 (31.4)	137 (4.6)	460 (15.5)		
Did your study returned to normal status									
No recovery	3374 (37.6)	5603 (62.4)	1240.442	0.000	1875 (20.9)	227 (2.5)	1272 (14.2)	135.505	0.000
Partial recovery	14878 (26.2)	41883 (73.8)			7165 (12.6)	759 (1.3)	6954 (12.3)		
Full recovery	3040 (17.7)	14095 (82.3)			1342 (7.8)	137 (0.8)	1561 (9.1)		
Have you actively learned about mental health related knowledge after the pandemic									
Yes	12333 (24.4)	38122 (75.6)	105.354	0.000	5896 (11.7)	573 (1.1)	5864 (11.6)	43.347	0.000
No	8959 (27.6)	23459 (72.4)			4486 (13.8)	550 (1.7)	3923 (12.1)		
Does the pandemic have an impact on your further study and employment									
No impact	4094 (19.9)	16429 (80.1)	786.477	0.000	1959 (9.5)	188 (0.9)	1947 (9.5)	81.566	0.000
Minor impact	8634 (25.3)	25480 (74.7)			4033 (11.8)	434 (1.3)	4167 (12.2)		
Significant impact	4753 (30.9)	10607 (69.1)			2369 (15.4)	261 (1.7)	2123 (13.8)		
Serious impact	1457 (35.3)	2667 (64.7)			801 (19.4)	103 (2.5)	553 (13.4)		
Unclear	2354 (26.9)	6398 (73.1)			1220 (13.9)	137 (1.6)	997 (11.4)		
Worry about being infected									
Not worried	6167 (29.3)	14889 (70.7)	316.540	0.000	3161 (15.0)	372 (1.8)	2634 (12.5)	86.429	0.000
A little worried	11642 (23.6)	37629 (76.4)			5430 (11.0)	547 (1.1)	5665 (11.5)		
Quite worried	2345 (26.3)	6580 (73.7)			1181 (13.2)	130 (1.5)	1034 (11.6)		
Extremely worried	1138 (31.4)	2483 (68.6)			610 (16.8)	74 (2.0)	454 (12.5)		
Depression Symptoms									
No depression	7390 (14.4)	43919 (85.6)	10543.520	0.000	2400 (4.7)	170 (0.3)	4820 (9.4)	2709.652	0.000
Mild depression	6339 (35.6)	11453 (64.4)			3105 (17.5)	231 (1.3)	3003 (16.9)		
Moderate depression	3508 (52.6)	3166 (47.4)			2123 (31.8)	213 (3.2)	1172 (17.6)		
Severe depression	4055 (57.1)	3043 (42.9)			2754 (38.8)	509 (7.2)	792 (11.2)		
Anxiety Symptoms									
No anxiety	9324 (16.5)	47158 (83.5)	9000.168	0.000	3271 (5.8)	263 (0.5)	5790 (10.3)	2596.000	0.000
Mild anxiety	6756 (40.0)	10154 (60.0)			3683 (21.8)	272 (1.6)	2801 (16.6)		
Moderate anxiety	3285 (48.9)	3433 (51.1)			2142 (31.9)	255 (3.8)	888 (13.2)		
Severe anxiety	1927 (69.7)	836 (30.3)			1286 (46.5)	333 (12.1)	308 (11.1)		
Social support									
Low-level	1573 (36.9)	2691 (63.1)	345.745	0.000	1001 (23.5)	94 (2.2)	478 (11.2)	583.725	0.000
Medium-level	19405 (25.3)	57438 (74.7)			9242 (12.0)	1015 (1.3)	9148 (11.9)		
High-level	314 (17.8)	1452 (82.2)			139 (7.9)	14 (0.8)	161 (9.1)		

### Risk factors associated with lifetime suicidal ideation

**Figure 1:**
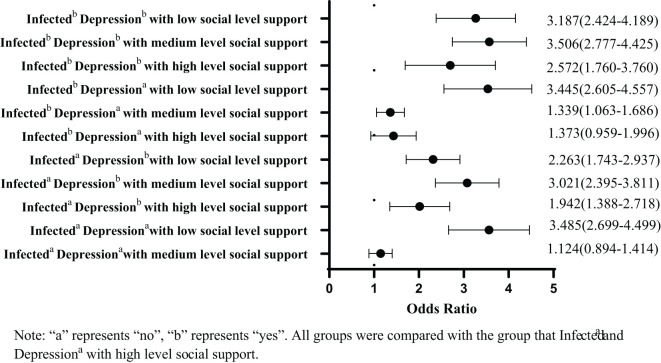
The superimposed effect of COVID-19 infection, depressive symptoms, social support on lifetime suicidal ideation.

**Table 3** shows the results of binary logistic regression analysis of risk factors associated with lifetime suicidal ideation. Being female, middle and high school students, from urban area, boarder at school, in a state of love, with higher family income levels, and with divorced or remarried parents were significantly associated with participants’ lifetime suicide ideation (*P*<0.05). Compared to authoritarian parenting styles, participants with autocratic, neglectful, and submissive parenting styles were significantly more likely to have lifetime suicidal ideation (*P*<0.05). Participants who were quarantined for longer periods of time by the COVID-19 had significantly higher risk for lifetime suicidal ideation (*P*<0.05). Participants who were not satisfied with their studies and felt impact of COVID-9 pandemic on their further education and employment were more likely to have suicidal ideation (*P*<0.05). After the lifting of COVID-19 restrictions, compared with participants who extremely worried about infection, participants who did not worry about infection at all had significantly higher rate of suicidal ideation (*P*<0.05). Participants with anxiety symptoms had at least 1.5 times higher rates of suicidal ideation than those without anxiety symptoms (OR, 1.530 [95% CI, 1.450–1.614] for mild anxiety, 1.999 [95% CI, 1.867–2.141] for moderate anxiety, and 4.321 [95% CI, 3.915–4.769] for severe anxiety). Compared with participants without COVID-19 infection and depression but with high level of social support, those with symptoms of infection and depression, as well as moderate levels of social support (OR, 3.506 [95% CI, 2.777–4.425) had the highest risk of lifetime suicidal ideation (**Figure 1**).

**Table 3:** Multivariable Regression Analysis of Risk Factors Associated with Lifetime Suicidal Ideation.

Variables	β	S_β_	Wald χ^2^	OR	95% CI	*P*
Gender						
Male					1 [Reference]	
Female	0.611	0.019	986.346	1.842	1.773–1.913	0.000
Registered residence						
Rural area					1 [Reference]	
Urban area	0.161	0.023	49.794	1.175	1.123–1.229	0.000
School type						
College or university					1 [Reference]	
Middle school	0.101	0.028	13.254	1.106	1.048–1.168	0.000
High school	0.122	0.023	27.904	1.130	1.080–1.182	0.000
Accommodation Type						
Day pupil					1 [Reference]	
Boarders at school	0.097	0.028	11.969	1.102	1.043–1.164	0.001
Other	0.061	0.099	0.385	1.063	0.876–1.290	0.535
Love situation						
Not in a romantic relationship					1 [Reference]	
Having a boyfriend	0.177	0.038	21.543	1.193	1.107–1.286	0.000
Having a girlfriend	0.098	0.048	4.128	1.103	1.003–1.213	0.042
Other	0.192	0.100	3.701	1.212	0.996–1.474	0.054
Family economic level						
Below local average					1 [Reference]	
Local average	0.292	0.060	23.613	1.339	1.190–1.507	0.000
Higher than local average	-0.009	0.019	0.212	0.991	0.954–1.029	0.645
Parents’ marital status						
Unmarried					1 [Reference]	
Married	0.008	0.067	0.013	1.008	0.884–1.148	0.909
Divorce	0.174	0.072	5.909	1.190	1.034–1.369	0.015
Remarry	0.164	0.073	5.029	1.179	1.021–1.361	0.025
Other	0.271	0.091	8.906	1.312	1.098–1.567	0.003
Parenting styles						
Authoritative pattern					1 [Reference]	
Autocratic pattern	0.366	0.022	284.514	1.442	1.382–1.505	0.000
Neglectful pattern	0.586	0.032	338.920	1.797	1.688–1.912	0.000
Submissive pattern	0.204	0.025	64.585	1.227	1.167–1.289	0.000
Quarantine time						
0 days					1 [Reference]	
<7 days	0.051	0.026	3.780	1.053	1.000–1.108	0.052
7–14 days	0.122	0.026	21.557	1.130	1.073–1.189	0.000
>14 days	0.383	0.042	81.797	1.467	1.350–1.594	0.000
How did you study at home during the pandemic?						
Regular learning					1 [Reference]	
Occasional learning	0.096	0.019	24.866	1.101	1.060–1.143	0.000
No learning	0.139	0.052	7.242	1.149	1.038–1.271	0.007
Academic during the COVID-19						
Academic progress					1 [Reference]	
Almost no impact	0.309	0.028	118.137	1.362	1.288–1.440	0.000
Academic decline	0.290	0.030	95.940	1.337	1.261–1.416	0.000
Academic satisfaction						
Extremely satisfied					1 [Reference]	
Satisfied	-0.117	0.046	6.413	0.889	0.812–0.974	0.011
General Satisfied	0.100	0.043	5.503	1.105	1.017–1.202	0.019
Dissatisfied	0.318	0.047	45.458	1.375	1.253–1.508	0.000
Extremely dissatisfied	0.550	0.060	84.913	1.734	1.542–1.949	0.000
Did your study returned to normal status						
Full recovery					1 [Reference]	
Partial recovery	0.054	0.026	4.417	1.056	1.004–1.110	0.036
No recovery	0.094	0.036	6.977	1.099	1.025–1.178	0.008
Have you actively learned about mental health related knowledge after the pandemic						
Yes					1 [Reference]	
No	0.053	0.018	8.278	1.055	1.017–1.093	0.004
Does the pandemic have an impact on your further study and employment						
No impact					1 [Reference]	
Minor impact	0.100	0.025	16.657	1.105	1.053–1.160	0.000
Significant impact	0.144	0.029	24.627	1.155	1.091–1.222	0.000
Serious impact	0.094	0.044	4.694	1.099	1.009–1.197	0.030
Unclear	0.050	0.033	2.263	1.051	0.985–1.122	0.132
Worry about being infected						
Extremely worried					1 [Reference]	
Quite worried	-0.033	0.048	0.472	0.967	0.880–1.064	0.492
A little worried	0.028	0.043	0.425	1.028	0.946–1.118	0.515
Not worried	0.380	0.045	72.672	1.462	1.340–1.595	0.000
Anxiety Symptoms						
No anxiety					1 [Reference]	
Mild anxiety	0.425	0.027	240.647	1.530	1.450–1.614	0.000
Moderate anxiety	0.693	0.035	393.598	1.999	1.867–2.141	0.000
Severe anxiety	1.464	0.050	846.128	4.321	3.915–4.769	0.000

### Risk factors associated with 12-month suicidal ideation

**Table 4** shows the results of multifactorial logistic regression analysis of risk factors associated with 12-month suicidal ideation. Participants’ suicidal ideation in the past 12 months was significantly associated with female (OR: 1.331, *P* = 0.000), middle school (OR: 2.354, *P* = 0.000), high school (OR: 1.708, *P* = 0.000), in love status (have a boyfriend: OR: 1.182, *P* = 0.007), parenting styles (autocratic: OR: 1.180, *P* = 0.000; neglectful: OR: 1.273, *P* = 0.000, and submissive: OR: 1.337, *P* = 0.000), quarantined time (7–14 days: OR: 1.146, *P* = 0.002), partial back to normal study status (OR:1.111, *P* = 0.023), not worried about infection of COVID-19 (OR: 1.225, *P* = 0.006), more depressive symptoms (mild: OR: 1.667, *P* = 0.000; moderate: OR: 2.423, *P* = 0.000; severe: OR: 3.595, *P* = 0.000), more anxiety symptoms (mild: OR: 1.506, *P* = 0.000; moderate: OR: 1.708, *P* = 0.000; severe: OR: 2.165, *P* = 0.000), and lower social support (low-level: OR: 2.224, *P* = 0.000).

**Table 4:** Multivariable Regression Analysis of Risk Factors Associated With 12-month Suicidal Ideation and Suicide Attempts.

Variables	12-month of Suicidal Ideation			12-month of Suicide Attempts		
	OR	95% CI	*P*	OR	95% CI	*P*
Gender						
Male	1 [Reference]					
Female	1.331	1.244–1.424	0.000	1.814	1.552–2.120	0.000
School type						
College or university	1 [Reference]					
Middle school	2.354	2.139–2.589	0.000	5.531	4.431–6.904	0.000
High school	1.708	1.582–1.844	0.000	2.469	2.020–3.019	0.000
Accommodation Type						
Day pupil	1 [Reference]					
Boarders at school	0.970	0.880–1.068	0.533	0.962	0.794–1.164	0.689
Other	0.913	0.649–1.283	0.599	1.764	0.962–3.233	0.066
Love situation						
Not in a romantic relationship	1 [Reference]					
Having a boyfriend	1.182	1.046–1.336	0.007	1.653	1.269–2.154	0.000
Having a girlfriend	1.174	0.993–1.388	0.060	1.269	1.586–3.095	0.000
Other	1.029	0.741–1.429	0.865	2.694	1.663–4.365	0.000
Parents’ marital status						
Unmarried	1 [Reference]					
Married	0.797	0.635–0.999	0.049	0.935	0.606–1.640	0.794
Divorce	0.901	0.708–1.147	0.398	1.086	0.688–1.969	0.761
Remarry	0.960	0.750–1.230	0.747	1.266	0.806–2.323	0.389
Other	0.995	0.736–1.345	0.973	1.461	0.783–2.726	0.233
Parenting styles						
Authoritative pattern	1 [Reference]					
Autocratic pattern	1.180	1.081–1.287	0.000	1.177	0.967–1.432	0.105
Neglective pattern	1.273	1.183–1.369	0.000	1.276	1.087–1.497	0.003
Submissive pattern	1.337	1.209–1.478	0.000	1.506	1.230–1.844	0.000
Have you been infected with COVID-19						
No	1 [Reference]					
Yes	1.038	0.977–1.104	0.226	1.042	0.912–1.191	0.545
Quarantine time						
0 days	1 [Reference]					
<7 days	1.039	0.951–1.136	0.396	1.038	0.851–1.266	0.715
7–14 days	1.146	1.050–1.251	0.002	1.128	0.931–1.367	0.220
>14 days	1.013	0.885–1.160	0.852	1.328	1.018–1.732	0.037
How did you study at home during the pandemic						
Regular learning	1 [Reference]					
Occasional learning	0.969	0.907–1.034	0.338	1.051	0.911–1.212	0.497
No learning	1.001	0.849–1.179	0.994	0.789	0.555–1.123	0.188
Academic satisfaction						
Extremely satisfied	1 [Reference]					
Satisfied	0.739	0.624–0.875	0.000	0.998	0.651–1.530	0.993
General Satisfied	0.765	0.659–0.889	0.000	1.080	0.741–1.575	0.689
Dissatisfied	0.775	0.661–0.909	0.002	1.050	0.709–1.555	0.806
Extremely dissatisfied	0.853	0.705–1.033	0.104	1.149	0.750–1.759	0.524
Did your study returned to normal status						
Full recovery	1 [Reference]					
Partial recovery	1.111	1.105–1.217	0.023	1.043	0.807–1.349	0.748
No recovery	1.120	0.995–1.262	0.061	1.055	0.858–1.298	0.611
Have you actively learned about mental health related knowledge after the epidemic						
Yes	1 [Reference]					
No	1.001	0.940–1.066	0.982	1.214	1.061–1.389	0.005
Does the pandemic have an impact on your further study and employment						
No impact	1 [Reference]					
Minor impact	0.893	0.805–0.990	0.031	0.997	0.801–1.241	0.979
Significant impact	0.926	0.828–1.036	0.181	0.994	0.785–1.258	0.958
Serious impact	0.981	0.843–1.143	0.809	1.028	0.761–1.389	0.856
Unclear	0.960	0.856–1.076	0.486	0.913	0.712–1.170	0.470
Worry about being infected						
Extremely worried	1 [Reference]					
Quite worried	1.050	0.894–1.234	0.550	1.142	0.824–1.584	0.425
A little worried	1.003	0.871–1.155	0.962	1.043	0.786–1.384	0.769
Not worried	1.225	1.059–1.418	0.006	1.434	1.073–1.916	0.001
Depression Symptoms						
No depression	1 [Reference]					
Mild depression	1.667	1.526–1.820	0.000	1.953	1.535–2.485	0.000
Moderate depression	2.423	2.161–2.717	0.000	3.449	2.603–4.570	0.000
Severe depression	3.595	3.108–4.158	0.000	6.679	4.889–9.126	0.000
Anxiety Symptoms						
No anxiety	1 [Reference]					
Mild anxiety	1.506	1.381–1.642	0.000	1.106	0.886–1.381	0.374
Moderate anxiety	1.708	1.501–1.942	0.000	1.603	1.216–2.115	0.001
Severe anxiety	2.165	1.812–2.587	0.000	3.864	2.831–5.273	0.000
Social support						
High level	1 [Reference]					
Medium level	1.030	0.807–1.315	0.811	0.997	0.563–1.764	0.992
Low-level	2.224	1.695–2.919	0.000	1.621	0.874–3.006	0.126

### Risk factors associated with 12-month suicide attempts

**Table 4** shows the results of multifactorial logistic regression analysis of risk factors associated with 12-month suicide attempts. Participants’ suicide attempts in the past 12 months were significantly associated with female (OR: 1.814, *P* = 0.000), middle school (OR: 5.531, *P* = 0.000), high school (OR: 2.469, *P* = 0.000), in love status (have a boyfriend: OR: 1.653, *P* = 0.000; have a girlfriend: OR: 1.269, *P* = 0.000), parenting styles (neglectful: OR: 1.276, *P* = 0.003; submissive: OR: 1.506, *P* = 0.000);, quarantined time (over 14 days: OR: 1.328, *P* = 0.037), without active learning mental health knowledge during the COVID-19 epidemic (OR: 1.214, *P* = 0.005), not worried about infection of COVID-19 (OR: 1.434, *P* = 0.001), more depressive symptoms (mild: OR: 1.953, *P* = 0.000; moderate: OR: 3.449, *P* = 0.000; severe: OR: 6.679, *P* = 0.000), and more anxiety symptoms (moderate: OR: 1.603, *P* = 0.001; severe: OR: 3.864, *P* = 0.000).

## Discussion

This appears to be the first study to simultaneously survey students in China across various developmental stages from middle school to university to examine the prevalence of suicidal ideation and suicide attempts assessed after the lifting of COVID-19 restrictions across the country. We found that 21292 (25.7%) students reported lifetime suicidal ideation, 10382 (12.5%) students reported 12-month suicidal ideation, and 1123 (1.4%) students reported suicide attempts, which is consistent with previous studies on the prevalence of suicidal ideation during the COVID-19 pandemic (38–40). According to a global meta-analysis result (41), the prevalence for lifetime suicidal ideation, suicide attempts were 15.1% and 2.6%, so we found that the prevalence of suicide ideation was higher, but the prevalence of suicide attempts has decreased. Our research findings were similar to those of a previous study (42), and the higher prevalence of suicide attempts during the pandemic may reflect pandemic-related stressors such as fear of illness, life changes, loneliness and decreased social support, which can harm mental well-being of adolescents (42).

In our sample, the prevalence of suicidal ideation was significantly higher among students in high schools (28.9%) than among those in colleges or universities (25.4%) or in middle schools (21.2%). Students at middle and high schools were at 1.7–5.5 times the risk of having thought about suicide or attempting it within the previous 12 months. This may reflect their physiological and psychological immaturity compared to young adults, as well as their more impulsive behavior patterns (43). It may also reflect stronger feelings of unhappiness, worthlessness, or fear that younger students felt in response to more than two years of social isolation during the pandemic (44), which affected them during a developmental period when parents and teachers are particularly likely to pressure young people academically because of the widespread belief that “academic performance in school is more important than anything else” (45). The results of this study showed that students who were less satisfied with their studies were more likely to think about suicide. In general, students may face myriad issues related to their studies, such as ongoing academic problems, social difficulties, missed coursework, and extensive absences (46). Adolescents may worry about managing issues at school and acclimating to school routines (47). Adolescents who experience strong psychological stress because of poor academic performance and intensive courses may be at higher risk of suicidal behavior (48). Indeed, stress and sleep disorders were more prevalent among adolescents than among adults in Italy (64.3% vs. 43.1%) (49).

Young students need to develop a stable adult identity, harmonious interpersonal relationships and capabilities to confront and act on reality (50). If students can develop these capabilities, they may appropriately adjust to personal crises and reduce their risk of suicidal behavior (50). Younger students are at particularly high risk of suicidal behavior if they exercise maladaptive coping skills in the face of persistent academic stress and negative emotions (51). Educational pressure on students decreases gradually as they approach adulthood, and students in college and university tend to have more mature coping skills than those in middle and high schools and this may lower their risk of suicidal behavior (52). These factors may help older students weather pandemic-related stresses such as social restrictions and interruptions or alterations in studies, which contribute to depression and sleep disorders among college students (53). So, the above are possible reasons why middle school students have a higher risk of suicidal ideation and intention than college students.

In this study, suicidal ideation and suicide attempts were significantly associated with being female, living in an urban area, boarding at school, having parents who exercise a non-authoritative parenting style, or having parents who have divorced or remarried. These factors have also been linked to suicidal ideation and suicide attempts in previous studies (54–60). However, students reporting higher household income level were at higher risk of suicide ideation and attempts within the previous 12 months than those with lower household income, which contrasts with studies from South Korea (61, 62). The adolescents from families with lower socioeconomic status may have been at higher risk because they were less likely to receive social and psychological resources and support from their family (63). The link between higher risk and higher household income in our study may reflect that adolescents who grow up in more affluent families are more likely to experience psychological neglect, receive little parental care or be rejected, controlled or overprotected by their parents (64). It is also possible that in families with higher economic levels, parents have higher expectations for education, and students feel worried and helpless due to the learning losses caused by the epidemic, leading to suicide (65).

Just over 40% of our study participants reported having been infected with SARS-CoV-2 after the lifting of COVID-19 restrictions, and they were at significantly higher risk of ever having thought about suicide than those who reported not been infected. This is consistent with the idea that fear and concern about COVID-19 infection can exacerbate psychological problems (66). For example, Krygsman et al. (2023) found that in a young Canadian adult sample that increased fear of COVID-19 was associated with greater generalized anxiety and somatization (67). Nevertheless, history of COVID-19 infection did not significantly affect risk of suicidal ideation or suicide attempts during the previous 12 months, which may reflect less distress given the widely reported efficacy of vaccines against the virus and the lower morbidity and mortality associated with later virus strains (68). Students in our sample who had been quarantined for longer periods were at higher risk of suicidal ideation, which may be related to increased loneliness and decreased social support (42).

In our sample, 38.1% of participants reported symptoms of depression and 31.8% reported symptoms of anxiety. Such symptoms correlated significantly with suicidal ideation and suicide attempts, and more severe symptoms were associated with higher risk, which is consistent with previous studies (69, 70). Depression and anxiety symptoms are the most frequent mental health problems among adolescents and are significant risk factors for suicide (71, 72). Among adolescents, depression, anxiety and other mental health problems may have worsened during the pandemic, increasing the prevalence of suicide (73). Our work highlights that prevalence of suicidal ideation and suicide attempts remains relatively high among young people even after the lifting of COVID-19 restrictions. Possible reasons may include relatively high rates of SARS-CoV-2 infection and high levels of psychological stress.

Only 2.1% of participants in our study indicated that they enjoyed a high level of social support, and our analysis linked low social support to significantly higher risk of thinking about or attempting suicide in the previous 12 months, which is consistent with previous studies (74, 75). The interpersonal psychological theory of suicidal behavior suggests that isolation increases the likelihood of suicide, and that perceived social support helps reduce or prevent feelings of isolation (76). Supportive relationships can mitigate suicidal ideation among adolescents and young adults (77). Further studies should be conducted to explore the relationship between types of social support and suicidal behavior.

Our analysis detected a joint influence of COVID-19 infection, depressive symptoms and social support on the risk of ever having thought about suicide in our sample. Compared to those who had not been infected, did not have depression symptoms and reported high social support, participants with COVID-19 infection, depression, and low level of social support had 2.2 to 3.5 times higher risk of lifetime suicidal ideation. Participants with COVID-19 infection, depression, and high level of social support had 1.9–2.5 times higher risk of lifetime suicidal ideation. The COVID-19 infection and depression symptoms may have been associated with a lower level of social support (78) and an increased risk of suicidal ideation. Strengthening social support from family, school and peers may mitigate the negative emotions caused by COVID-19 infection and depressive symptoms, thereby reducing risk of suicidal ideation and suicide attempts.

Our study, together with a growing global literature, argue for prioritizing the development of prevention programs targeting children and adolescents, among whom suicide rates are increasing. Prevention strategies may include limiting access to means of suicide (e.g., limiting access to guns, knives and sedatives), collaborating with media to encourage responsible reporting on suicide, promoting healthy socio-emotional life skills among adolescents, and establishing programs geared toward early identification, assessment, management, and follow-up of individuals affected by suicidal behavior (45). Dialectical cognitive therapy and psychodynamic therapy may be effective treatments for young people who have been thinking about suicide or have attempted it (79, 80).

### Strengths and limitations

The strengths of this study include its relatively large sample of 162 public and private educational institutions in urban and rural regions that spanned the full range of adolescent development, from middle school through university. To the best of our knowledge, this is the first study to systematically investigate the prevalence and risk factors of suicidal ideation and suicide attempts among young people from 12 to 24 years old after the lifting of COVID-19 restrictions. Our findings may help guide policy making, recognition of high-risk populations, and the design of frameworks for managing psychological crises in specific populations and intervening in severe public health events such as pandemics.

At the same time, this study has several limitations. First, it involved an online survey of a convenience sample from one province in China. Whether our results can be generalized to other parts of China or to other countries remains unclear. Second, our data came entirely from self-report, increasing the risk of under- or overreporting due to subjective factors in participants that might have biased their responses, such as feelings of guilt or shame. Third, this was a cross-sectional study, which prevents causal analyses. Establishing causation in the absence of an experimental design with randomization is unlikely, and in the case of suicidality, would be unethical. Longitudinal follow-up studies should be conducted to explore whether the associated factors that we identified here can cause suicidal ideation or suicide attempts.

## Conclusions

After the lifting of COVID-19 restrictions, the prevalence of lifetime suicidal ideation was 25.7%, the 12-month prevalence of suicide ideation was 12.5%, and the 12-month prevalence of suicide attempts was 1.4% among students in China. The associated factors of suicidal ideation and attempts among adolescent and youth students included being female, urban registered residence, middle school and high school, boarders at school, in love status, higher family income levels, divorced or remarried parents, specific family parenting patterns (e.g., autocratic, neglective or submissive pattern), COVID-19 infection, quarantined for longer periods of time, not worried about COVID-19 infection after the lifting of COVID-19 restrictions, unsatisfactory learning, with much more impact on further study and employment, depression and anxiety symptoms, and low-level of social support. Targeted, specific psychosocial prevention and interventions are urgently needed to meet the mental health demands of students.

## Data availability statement

The original contributions presented in the study are included in the article/supplementary material. Further inquiries can be directed to the corresponding authors.

## Ethics statement

The studies involving humans were approved by Biomedical Research Ethics Committee, West China Hospital of Sichuan University (No: 2022-1790). The studies were conducted in accordance with the local legislation and institutional requirements. Written informed consent for participation in this study was provided by the participants’ legal guardians/next of kin.

## Author contributions

QX: Conceptualization, Data curation, Formal analysis, Investigation, Writing – original draft, Writing – review & editing. XH: Conceptualization, Data curation, Investigation, Writing – review & editing. JY: Conceptualization, Data curation, Formal analysis, Investigation, Writing – original draft. YM: Conceptualization, Data curation, Formal analysis, Investigation, Writing – original draft. CW: Conceptualization, Data curation, Formal analysis, Investigation, Writing – original draft. ZD: Conceptualization, Data curation, Formal analysis, Investigation, Writing – original draft. JC: Conceptualization, Data curation, Formal analysis, Investigation, Writing – original draft. AD: Data curation, Investigation, Writing – original draft. WT: Conceptualization, Data curation, Investigation, Writing – original draft. XC: Conceptualization, Data curation, Investigation, Writing – original draft. WS: Data curation, Investigation, Writing – original draft. YJ: Data curation, Investigation, Writing – original draft. JX: Data curation, Investigation, Writing – original draft. LY: Conceptualization, Data curation, Investigation, Writing – original draft. YH: Conceptualization, Data curation, Investigation, Writing – original draft. WZ: Conceptualization, Data curation, Investigation, Writing – review & editing. MR: Conceptualization, Data curation, Formal analysis, Funding acquisition, Investigation, Supervision, Writing – original draft, Writing – review & editing.
